# The predictive effect of fear-avoidance beliefs on low back pain among newly qualified health care workers with and without previous low back pain: a prospective cohort study

**DOI:** 10.1186/1471-2474-10-117

**Published:** 2009-09-24

**Authors:** Jette Nygaard Jensen, Karen Albertsen, Vilhelm Borg, Kirsten Nabe-Nielsen

**Affiliations:** 1 The National Research Centre for the Working Environment, Copenhagen, Denmark

## Abstract

**Background:**

Health care workers have a high prevalence of low back pain (LBP). Although physical exposures in the working environment are linked to an increased risk of LBP, it has been suggested that individual coping strategies, for example fear-avoidance beliefs, could also be important in the development and maintenance of LBP. Accordingly, the main objective of this study was to examine (1) the association between physical work load and LBP, (2) the predictive effect of fear-avoidance beliefs on the development of LBP, and (3) the moderating effect of fear-avoidance beliefs on the association between physical work load and LBP among cases with and without previous LBP.

**Methods:**

A questionnaire survey among 5696 newly qualified health care workers who completed a baseline questionnaire shortly before completing their education and a follow-up questionnaire 12 months later. Participants were selected on the following criteria: (a) being female, (b) working in the health care sector (n = 2677). Multinomial logistic regression analysis was used to evaluate the effect of physical work load and fear-avoidance beliefs on the severity of LBP.

**Results:**

For those with previous LBP, physical work load has an importance, but not among those without previous LBP. In relation to fear-avoidance beliefs, there is a positive relation between it and LBP of than 30 days in both groups, i.e. those without and with previous LBP. No moderating effect of fear-avoidance beliefs on the association between physical work load and LBP was found among cases with and without LBP.

**Conclusion:**

Both physical work load and fear-avoidance beliefs matters in those with previous LBP. Only fear-avoidance beliefs matters in those without previous LBP. The study did not find a moderating effect of fear-avoidance beliefs on the association between physical work load and LBP.

## Background

The prevalence of low back pain (LBP) among health care workers is high; studies have found that the 12-month prevalence is between 45% and 63% [1-5] compared with 40-50% in the general population among employees in general [6]. When health care students (health care helpers and assistants) begin their studies they do not have a higher prevalence of LBP than that of the same age range in the general Danish population[7]. Among 5700 health care students in Denmark, 51% reported LBP trouble at the end of their education. However, this prevalence increased to 65% one year later when they were in employment as health care workers [8]. This difference between those under education and those in employment indicates that the high prevalence of LBP may be caused by factors experienced in the job.

Several well known LBP risk factors are present when working with care of the elderly: physical strain, such as heavy manual work; twisting and bending; standing in forward-bent and twisted postures; poor ergonomic/lifting conditions; and frequent positioning of bedridden patients [9-11]. Previous studies among health care helpers and nurses have found that psychosocial work-related factors were associated with LBP [9,10,12-14]. However, a later review concluded that the evidence regarding the effect of the psychosocial work environment on LBP were low because of methodological problems [15].

Research suggests that a person's coping style and beliefs about pain may be relevant in understanding its development [16]. In a study of young health care workers and distribution workers without LBP, it was found that physical work load was predictive of LBP, but also pain related fear was found to be important. In the development of LBP in acute and chronic cases, studies have suggested that fear-avoidance beliefs are an influential psychological factor [17-20], and there is some evidence that suggest that fear-avoidance may play a role when pain becomes chronic, but little evidence with regard to early stages of LBP [21]. Fear-avoidance beliefs refer to the fear-induced avoidance of movements or activities that are expected to be painful. These beliefs contributes to the development of musculoskeletal pain into a chronic pain syndrome [22,23]. In theory, the response to pain can be seen as a continuum with two extremes: confrontation and avoidance [24]. A person who displays confrontational behaviour will strive to return to the normal level of activity, despite the pain. On the contrary, a person with avoidance behaviour will avoid those activities that are expected to cause increased pain, e.g. physical activity. This behaviour is believed to increase the risk of developing chronic pain [18,25,26] and is not in accordance with guidelines based on LBP research, which recommend LBP treatment by staying active and continuing normal daily life, including going to work [27,28].

Nearly all research on fear-avoidance beliefs to date has been conducted on chronic LBP patients, except a few studies focusing on acute LBP and fear-avoidance belief. These last mentioned studies found that high levels of fear-avoidance beliefs were present already in the early stages of LBP [18,20,29]. To our knowledge, only Linton and Buer [30] have studied fear-avoidance beliefs in a pain-free general population. They concluded that pain-free people with high fear-avoidance beliefs had a higher risk of having an episode of back pain and lowered physical function at follow-up one year later.

In summary, LBP is highly frequent among health care helpers, and high physical work load is considered a risk factor. There seems to be moderate evidence showing that high fear-avoidance is positively associated with LBP mainly among hospitalized LBP patients. However, specific knowledge is lacking as to how fear-avoidance beliefs develop and how it is involved in the development of LBP. For this purpose it is relevant to study fear-avoidance beliefs in non-patient and non-chronic populations. As previous history of LBP is a well-known predictor of later LBP [31], we believe that it is important to differentiate between employees with and without previous LBP experience. Thereby it is possible to study the process of maintenance of LBP and the process of developing LBP, respectively.

Based on a cohort of newly qualified female health care workers with and without previous LBP experience respectively, the aims of this study were to (a) examine the associations between physical work load and the development of LBP, (b) examine the predictive effect of fear-avoidance beliefs on the development of LBP, and (c) examine the moderating effect of fear-avoidance beliefs on the association between LBP and physical work load.

## Methods

### Design

Background: Data used in this study were from a national survey - the Danish Health Care Worker Cohort-Class of 2004 (DHCWC-2004), a prospective cohort study of all Danish health care helpers and assistants who qualified in 2004. The health care helpers undergo 14-19 months of education, depending on the length of their basic school education. The education of health care assistants (continuation of the health care helper education) takes an additional 20 months. This study consists of data from baseline (2004) and the one-year follow-up (2005). In this article both health care helpers and health care assistants are referred to as health care workers.

Baseline 2004: at baseline, 6329 health care workers were invited to participate; 5696 completed the baseline questionnaire (90%). The baseline survey took place shortly before the students qualified. The questionnaire was completed during class hours with a researcher from the research group present, ready to assist if needed.

**Figure 1:**
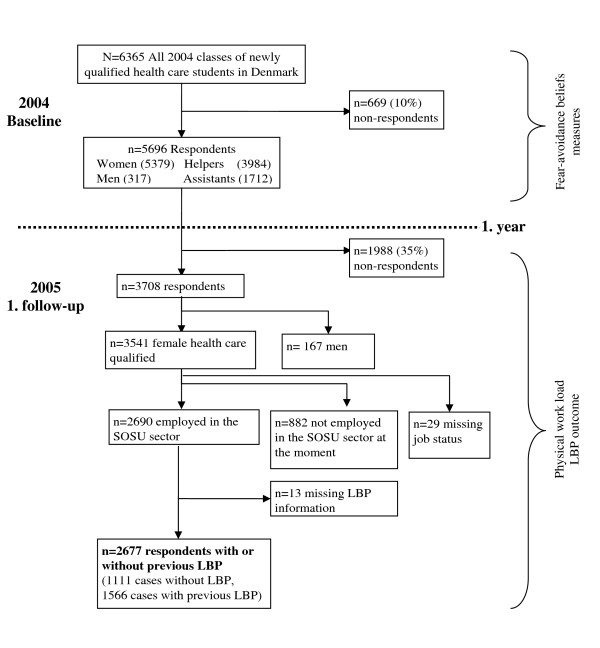
**Flowchart of the study population**.

Follow-up 2005: the follow-up questionnaire was posted to the participants 12-months after the baseline study, irrespective of whether they were working in the nursing or home care sector, whether they had found other areas of work, or whether they were continuing their education. Of the 5696 who completed the baseline questionnaire, 3708 (65%) completed and returned the one-year follow-up questionnaire - see figure 1.

The study has been notified to and registered by the Danish Data Protection Agency (Datatilsynet, see  for details). Questionnaire- and register-based studies do not need approval from the Danish National Committee on Biomedical Research Ethics (Den Centrale Videnskabetiske komité, see  for details).

### Outcome variable

Low back pain: information on LBP at follow-up was measured by using the question of 12-months prevalence from the Standardised Nordic Questionnaires for the analyses of musculoskeletal symptoms (SNQ) [32]: "What is the total length of time that you have had low back trouble during the last 12 months?" As in the original SNQ, the location of the lower back was defined by a drawing with a marked area. It was divided into 5 response categories: No trouble/1-7 days/8-30 days/more than 30 days, but not every day/every day. In the statistical analyses, the last two response categories were combined into one category ("more than 30 days").

### Determinants

Fear-avoidance beliefs: fear-avoidance beliefs at baseline were measured by the fear-avoidance beliefs questionnaire (FABQ) developed by Waddell et al. [23]. The questionnaire consisted of two subscales: fear-avoidance beliefs about work (FAB-work) and fear-avoidance beliefs about physical activity (FAB-physical activity). The scales are designed to measure the beliefs about how work and physical activity effect pain and to what extent these activities should be avoided. The items measuring fear-avoidance beliefs are mainly designed for work-experienced chronic patients. Our study population at baseline encompassed participants without a job the last 14-34 months, except for practical training during their education. The participants filled out the questionnaires just before they qualified and were informed - verbally - to think of practical training as their work when they filled out the questionnaire. Further, not all participants had had experiences with LBP, so contrary to the introduction to FABQ made by Waddell et al. (1993), who focused only on pain in the back, this study asked the respondents to state how their work and their physical activity affected pain in their back as well as in their neck and shoulders. Only healthcare students with musculoskeletal pain (neck, shoulder and upper or low back pain) during the previous 12 months were requested to answer the questions on fear-avoidance beliefs at the baseline measurement. The FAB-work and FAB-physical activity were measured with a shortened version of the FABQ (items number 1, 2, 3, 4, 12, 13, 14 in the original). Each item was scored on a rating scale from 0 to 6 points: 0 = disagree, 3 = unsure, and 6 = agree. An example of an item from the FAB-work scale is: "I should not do my normal work with my present pain". An example of an item from the FAB-physical activity scale is: "My pain was caused by physical activity". The questions were translated by experienced researchers and tested in a qualitative pilot study (N = 31).

Because FAB-physical activity and especially FAB-work had an asymmetric distribution (skewness 0.254 and 1.604, std.error of skewness 0.056 and 0.063, respectively), z-scores were computed for both and standardized so the mean was zero. Converting scores to a z-score is a way of standardizing them. High scores indicated increased levels of fear-avoidance beliefs. A factor analysis confirmed the 2-factor structure of the fear-avoidance beliefs questions and Cronbach's alpha for FAB-work and FAB-physical activity was 0.80 and 0.73, respectively.

Physical work load: physical work load at follow-up was measured as unfavourable postures of the body: bending, twisting, kneeling or squatting as well as handling heavy loads during work. Fourteen items were used to describe these postures. Three items described postures of the trunk: strongly inclined, twisted and laterally bent. Two items described positions of the arms: one arm above shoulder height, and two arms above shoulder height. Three items described the position of the legs: squatting, kneeling on one or both knees, walking or moving. Three items described lifting of weights with the trunk upright and three items described lifting with the trunk inclined 60 degrees: light (up to 10 kg.), medium (10- 20 kg.) and heavy (more than 20 kg.). The items were also presented as pictograms. The answers were given on a 5-point rating scale ranging from "never" to "very often" [33]. All the questions were transformed into a physical work load index developed and validated by Hollmann [33], where each question had a weighted value. These values were aggregated to one value which estimated the total load of the lumbar spine in the job. The scale was categorized into: low physical work load (0-11.17), medium physical work load (11.17-19.43), and high physical work load (19.43-52.56).

### Covariates

Age: age was categorised into 10-year intervals: ≤ 19 years of age, 20-29 years of age, 30-39 years of age, 40-49 years of age and > 50 years of age.

Leisure time physical activity: to measure leisure time physical activity we used a slightly modified version of the question from Saltin and Grimby [34]: " If you should describe your spare time physical activities including transport to work, which group below do you belong to?" in four response categories: almost physically passive < 2 hours/week, little physical activity 2-4 hours/week, fatiguing activity 2-4 hours/week, regularly strenuous hard training > 4 hours/week.

Smoking: smoking was assessed by asking "Do you smoke every day?" with three response categories: (1) yes, (2) no, but I have smoked before, (3) no, I have never smoked.

Body Mass Index: weight and height were reported in order to compute a BMI index. BMI < 18.4 kg/m2 = underweight, BMI 18.5-24.9 kg/m2 = normal weight, BMI 25.0-29.9 kg/m2 = overweight and BMI > 30.0 kg/m2 = obese.

Psychosocial work factors: psychosocial work factors were measured at follow-up by the Copenhagen Psychosocial Questionnaire (COPSOQ) [35]. The following scales were used to measure psychological dimensions in the job: influence at work, two items, (Cronbach's Alpha .73); social support, three items, (Cronbach's Alpha 0.82); meaning at work, three items, (Cronbach's Alpha 0.74); role clarity, two items, (Cronbach's Alpha 0.73); and role conflict, two items, (Cronbach's Alpha 0.46). Four single items measured emotional job demands: commitment to workplace, demands for hiding emotions, and predictability. There were five response categories to all scales and single items: always/often/sometimes/rarely/never or almost never.

### Study population

Of the 3708 respondents who returned the follow-up questionnaire, 2677 health care workers were included in present analyses - see figure 1. Inclusion criteria were: 1) female respondents (as they constituted 95% of the entire study population), 2) being employed as a health care worker at follow-up. Those who were without a job, in a job not involving health care, on long term sickness absence or under continued education at follow-up were excluded from the analysis as they could not answer the questions about their working environment. The remaining 2677 respondents were divided into two groups: the 1111 participants who did not have present or previous experiences with LBP at baseline were defined in this article as "cases without LBP" and the 1566 respondents with previous or present LBP experience at baseline were defined as "cases with previous LBP".

### Statistical analyses

SPSS 17.0 was used for statistical analysis (SPSS Chicago, USA)

Multinomial logistic regression analysis was conducted to estimate the association between physical work load and the number of days with LBP during the previous 12 months. Multinomial logistic regression is used when an outcome has more than two main categories. In the present study, LBP was categorized into four groups (0 days, 1-7 days, 8-30 days, >30 days) to investigate the main and moderating effect of fear avoidance. We used four levels of the outcome because categorizing LBP-cases into only one group regardless of the acuteness or chronicity of their LBP may cover a multitude of underlying conditions. In one epidemiologic study it was found that various variables associated differently with LBP and differed depending on the subdefinitions of LBP in the previous year [36]. By categorizing LBP into four groups we were able to explore whether fear-avoidance beliefs were differently related to different durations of LBP.

In a preliminary analysis we tested for significant associations between psychosocial work-related variables and LBP. The two psychosocial variables that reached significance were emotional job demands and influence at work (p ≤ 0.05). They were included in the main analysis together with individual variables, physical work load and fear-avoidance beliefs. Non-associated psychosocial factors were social support, meaning at work, role clarity, role conflicts, demands for hiding emotions and predictability (p ≥ 0.05).

The association between physical work load, fear-avoidance beliefs, and LBP and the moderating effects of fear-avoidance beliefs were estimated in the following three models: first (model A), we made two simple tests without covariates of: (1) FAB-physical activity and physical work load, and (2) FAB-work and physical work load to test for their associations with LBP. Second (model B), we tested for the same associations as in model A, but controlled for the individual factors: age, leisure time physical activity, smoking and body mass index and the psychosocial work-related factors emotional demands and influence at work. Third, (model C), we tested for a moderating effect by creating the interaction terms physical work load x FAB-work, and physical work load x FAB-physical activity. All analyses were stratified into two groups: cases without LBP and cases with previous LBP. The stratification was based on a question from the baseline questionnaire: "Have you ever had low back pain (pain or discomfort)?" There were two response categories: "Yes" (cases with previous LBP) or "No" (cases without LBP).

## Results

### Characteristic of study population

The average age at baseline was 35.7 years (SD 10.74) ranging from 18 to 60 years of age.

At the end of participants' education, the most prevalent musculoskeletal problem within the previous 12 months was neck pain (61.9%) among cases without LBP. Among cases with LBP, the most prevalent musculoskeletal problem during the previous 12 months was LBP (88.2%). During the 1-year follow-up period, 520 of the 1,111 (47%) who had never experienced LBP at baseline developed a LBP problem. The 520 new cases were categorized in 269 having LBP 1-7 days during the previous 12 months, 162 having LBP 8-30 days, and 89 having > 30 days. Characteristics of the sample are described in Table 1. The mean baseline scores for FAB-work at baseline were 16.86 (SD 23.9, n = 1082) and 12.96 (SD 22.28, n = 398) for cases with and without previous LBP, respectively. The mean baseline scores for FAB-physical activity at baseline were 39.66 (SD 25.5, n = 1338) and 33.31 (SD 26.6, n = 545) for cases with and without previous LBP, respectively.

**Table 1:** Characteristics of newly qualified health care workers with and without previous episodes of LBP at baseline

**Time at measurement**	**Variable**		**Cases without LBP (n = 1111)**	**%**	**Cases with previous LBP (n = 1566)**	**%**
Baseline	Age					
		< 20	68	6.1	91	5.8
		20-29	331	29.8	497	31.7
		30-39	309	27.8	437	27.9
		40-49	292	26.3	384	24.5
		>50	111	10	157	10
Baseline	Education					
		Health care helper	741	66.7	972	62.1
		Health care assistant	370	33.3	594	37.9
Baseline	Foreign background					
		Born in Denmark	962	86.6	1413	90.2
Baseline	Living status					
		Living with partner	131	12	141	9.1
		Living with spouse/partner				
			734	76	1058	75.2
Baseline	Children living at home					
		No	504	45.7	754	48.6
		1 child	213	19.3	289	18.6
		2 children	257	23.3	351	22.6
		3 or more children	128	11.6	159	10.2
Baseline	Musculoskeletal disorder previous 12 months					
		Low back	-	-	1349	88.2
		Upper back	230	20.8	584	37.8
		Neck	682	61.9	1052	67.5
		Shoulder	356	32.1	798	51.3
Follow-up	Days with low back pain previous 12 months					
		No pain	586	53	331	21.3
		1-7 days	269	24.3	404	26
		8-30 days	162	14.6	444	28.5
		> 30 days	89	8	377	24.3
Follow-up	Body mass index					
		< 18.5	19	3.2	27	2.8
		18.5-24.9	419	60	533	57.4
		25.0-29.9	188	27.3	270	27.7
		≥ 30	85	9.6	153	12.2
Follow-up	Smoking					
		Current smoker	429	38.9	682	43.9
		Ex-smoker	227	20.6	333	21.4
		Never smoked	446	40.5	538	34.6
Follow-up	Leisure time physical activity					
		Sedentary	77	7.1	137	8.9
		Light	570	52.4	778	50.5
		Moderate	377	34.7	540	35.1
		High	64	5.9	85	5.5

Note: Foreign background, living status and musculoskeletal disorder previous 12 months do not summarize to 100% as they were single items with the response categories yes/no

### Associations between physical work load and LBP

Among cases without previous LBP, the associations between physical work load and LBP were not significant, neither before nor after adjustment for individual factors and psychosocial work related factors (Model A and B, table 2). Among cases with previous LBP there were significant associations between physical work load and LBP both before and after adjustment. A dose-response relationship was found in both ways: higher physical work load was more strongly associated with LBP than were lower levels of physical work load and the higher the number of days with LBP, the higher the estimate. However, none of the steps were significant, in that the confidence intervals overlapped (Model A and B, tables 3).

**Table 2:** Associations between physical work load and fear-avoidance beliefs on LBP. Cases without previous LBP

		**1-7 days of LBP**			**8-30 days of LBP**			**> 30 days of LBP**		
		
**Model A**		**P-value**	**OR**	**CI 95%**	**P-value**	**OR**	**CI 95%**	**P-value**	**OR**	**CI 95%**
**Physical work load**	High	0.091	1.90	0.90-3.98	0.184	1.65	0.79-3.47	0.149	1.96	0.79-4.86
	Medium	0.579	1.23	0.59-2.60	0.531	1.26	0.61-2.61	0.183	0.46	0.15-1.43
	Low	-	-	-	-	-	-	-	-	-
	FAB-work	0.586	1.11	0.76-1.63	0.443	1.16	0.80-1.68	0.00	2.08	1.42-3.04
										
**Physical work load**	High	0.563	1.19	0.06-2.14	0.009	2.45	1.25-4.79	0.062	2.15	0.96-4.80
	Medium	0.922	1.03	0.58-1.84	0.058	1.91	0.98-3.76	0.828	0.90	0.35-2.30
	Low	-	-	-	-	-	-	-	-	-
	FAB-ph.activity	0.827	1.03	0.81-1.31	0.902	1.02	0.78-1.33	0.008	1.54	1.19-2.13
										
Model B										
**Physical work load**	High	0.261	1.61	0.70-3.67	0.483	1.33	0.60-2.98	0.255	1.82	0.64-5.10
	Medium	0.955	1.02	0.45-2.35	0.781	0.89	0.40-1.99	0.107	0.32	0.08-1.28
	Low	-	-	-	-	-	-	-	-	-
	FAB-work	0.711	1.08	0.71-1.65	0.66	1.10	0.72-1.68	0.00	2.26	1.45-3.51
										
**Physical work load**	High	0.737	1.12	0.59-2.12	0.098	1.85	0.89-3.82	0.165	1.88	0.77-4.57
	Medium	0.611	0.85	0.45-1.61	0.47	1.31	0.63-2.74	0.572	0.75	0.27-2.07
	Low	-	-	-	-	-	-	-	-	-
	FAB-ph.activity	0.565	1.08	0.83-1.42	0.724	1.06	0.78-1.42	0.005	1.66	1.17-2.36

Model A: No adjustments

Model B: Adjusted for age, smoking, leisure time physical activity, body mass index, emotional job demands and influence at work

**Table 3:** Associations between physical work load and fear-avoidance beliefs on LBP. Cases with previous LBP

		**1-7 days of LBP**			**8-30 days of LBP**			**> 30 days of LBP**		
		
**Model A**		**P-value**	**OR**	**CI 95%**	**P-value**	**OR**	**CI 95%**	**P-value**	**OR**	**CI 95%**
**Physical work load**	High	0.012	1.92	1.15-3.22	0.00	2.88	1.75-4.72	0.00	3.47	2.05-5.87
	Medium	0.01	1.93	1.17-3.16	0.004	2.07	1.27-3.40	0.00	2.80	1.67-4.72
	Low	-	-	-	-	-	-	-	-	-
	FAB-work	0.131	1.21	0.95-1.54	0.023	1.13	1.04-1.65	0.00	1.53	1.21-1.93
										
**Physical work load**	High	0.008	1.87	1.18-2.97	0.00	2.45	1.60-3.87	0.00	3.37	2.11-5.36
	Medium	0.03	1.98	1.27-3.10	0.003	1.93	1.25-3.00	0.00	2.77	1.75-4.41
	Low	-	-	-	-	-	-	-	-	-
	FAB-ph.activity	0.322	1.10	0.91-1.40	0.405	1.08	0.90-1.31	0.012	1.28	1.06-1.56
Model B										
**Physical work load**	High	0.045	1.76	1.01-3.07	0.00	2.93	1.70-5.06	0.00	3.08	1.74-5.45
	Medium	0.013	1.97	1.15-3.37	0.001	2.44	1.42-4.19	0.00	3.05	1.74-5.35
	Low	-	-	-	-	-	-	-	-	-
	FAB-work	0.104	1.24	0.96-1.61	0.038	1.31	1.02-1.69	0.00	1.59	1.23-2.04
										
**Physical work load**	High	0.072	1.59	0.96-2.62	0.00	2.41	1.48-3.95	0.00	2.90	1.74-4.83
	Medium	0.011	1.86	1.15-3.00	0.002	2.01	1.30-3.39	0.00	2.80	1.71-4.61
	Low	-	-	-	-	-	-	-	-	-
	FAB-ph.activity	0.734	1.04	0.84-1.28	0.584	1.06	0.86-1.30	0.03	1.26	1.02-1.55

Model A: No adjustments

Model B: Adjusted for age, smoking, leisure time physical activity, body mass index, emotional job demands and influence at work

### Predictive effect of fear-avoidance beliefs on low back pain

The adjusted values showed a positive association between fear-avoidance beliefs and LBP of more than 30 days in both groups, i.e. those without and with previous LBP. In addition, for those with previous LBP, there was a dose-response between numbers of days with LBP and fear-avoidance beliefs, although small steps in between the estimates (see table 2 and 3).

In other words, only fear-avoidance beliefs matters for the development of LBP in those without previous LBP, but both physical work load and fear-avoidance beliefs matters in those with previous LBP. However, for the latter group, the estimated odds ratio was higher for physical work load than for fear avoidance, regardless of the duration of LBP.

### Moderating effects of fear-avoidance beliefs on the association between low back pain and physical work load

No significant moderating effects were found of FAB-work or FAB-physical activity on the association between physical work load and LBP (results not shown).

## Discussion

This study aimed to enhance understanding of the established link between physical work load and LBP among health care workers by studying the predictive effect of fear-avoidance beliefs. The findings provide new information on the role of physical work load and fear-avoidance beliefs on LBP and, to a certain extent, support previous research. Three main findings are discussed: (1) the association between physical work load and LBP among cases with previous LBP, and the lack of association among cases without LBP; (2) the predictive effect of fear-avoidance beliefs on higher number of days with LBP; and (3) the lack of moderating effects of fear-avoidance beliefs.

First, the associations between physical work load and LBP were insignificant among cases without previous LBP but not among cases with previous LBP. Thus, those without LBP experiences currently seem to be less vulnerable to a high physical load. This might be because relatively healthy people can withstand higher work loads [37], while those who have already experienced LBP have a lower threshold level for physical exposure. The result differs somewhat from a cross-sectional study that found a significant association between physical work load and "first-ever low back pain" among young workers (mean age 22 years) in their first job (health care or distribution service) [38]. An explanation could be that respondents in our study were on average 36 years old when they qualified, and many had had several years of work experience, which again, could indicate a "healthy worker" effect or a protective effect of work experience.

Second, fear-avoidance beliefs had a predictive effect on LBP. Our results indicate that both types of fear-avoidance belief-FAB-work and FAB-physical activity-are prospectively associated with a higher number of days with LBP (30 days or more) in cases with and without previous LBP experience. Our results support previous studies that found associations between fear-avoidance beliefs and LBP [18-20,29], although we are unable to directly relate our results to other studies because study designs, aims and the definition of LBP differ from those in our study. Nevertheless, our results are comparable with a study by van Nieuwenhuyse et al. (2006), who studied young health care workers and distribution workers. Their results showed that physical work and high levels of pain-related fear were risk factors for developing LBP one year later among young workers [39].

Third, we did not find any moderating effect of fear-avoidance beliefs on the association between physical work load and LBP. Accordingly, it is not likely that fear-avoidance beliefs will increase the negative effect of physical work load on LBP.

Overall, our results support an independent predictive effect of fear-avoidance beliefs on serious or more or less chronic levels of LBP. We did not find an effect on short-term LBP (1-7 days). This is in accordance with two longitudinal studies by Sieben et al. (2002, 2005), who examined the role of fear-avoidance beliefs at early stages of LBP (≤ 3 weeks). Their results did not support a predictive effect of fear-avoidance on early stages of LBP, and they questioned the role of fear-avoidance on early stages of LBP. Linton et al. (2000) suggested that fear-avoidance beliefs already exist due to prior pain experiences, and that these beliefs are activated by pain and enhanced in a reciprocal process with the pain experience [30]. Our result cannot confirm nor disprove this hypothesis, but it may explain why cross-sectional studies or studies with relatively short follow-up periods have found an association between fear-avoidance beliefs and acute LBP. The non-significant effect of fear-avoidance beliefs on 1-7 days (short-term LBP) in our study could be because there are no high fear-avoidance beliefs *before *LBP episodes, but they are developed in an interaction process with the LBP experience. Furthermore, previous studies have found that fear-avoidance beliefs are higher among chronic LBP patients than among those with acute LBP [29], and in our study population, cases with previous LBP have a higher mean score on fear-avoidance beliefs than cases without LBP. This indicates that fear-avoidance beliefs not are static personal traits but are modifiable and depend on the experience (e.g. pain experience).

We did not find a moderating effect of fear-avoidance beliefs on the association between physical work load and LBP. It could be argued that a moderating effect would be easier to detect if the effect-variables were measured at the same time and not, as in this case, at baseline and follow-up, respectively, particularly if the level of fear-avoidance beliefs changes over time.

The Danish version of FABQ has not been validated. The FAB-work scale in this study had a high positive skewness indicating that about half of the participant had no or very low fear-avoidance beliefs about work. The participants filled out the questionnaires just before they qualified and had not been employed in the last 14 to 34 months, but only had practical training. All though the participants were informed - verbally - to think of the practical training as their work when they filled out the questionnaire, this message may very well not have been communicated effectively to all. Therefore participants may have underestimated their fear-avoidance beliefs, and the prevalence of participants with no or low fear-avoidance beliefs about work may be overestimated. If this has been the case, the effect of fear-avoidance beliefs may accordingly have been underestimated.

The role of distress and depression has been found to play a role on early stages of LBP due to social withdrawal and reduced activity [40]. Distress and depression may in fact be so intimately linked to LBP and to fear-avoidance beliefs that it might potentially be over control, if we adjusted for it. Therefore we have not included measures of distress or depression in our analyses. However, the possible relationships between depression, fear-avoidance beliefs and LBP may deserve specific attention in future research.

There might be a differential misclassification between participants with and without LBP symptoms. Persons with LBP may tend to assess their physical work load as more demanding than would those without [41]. Therefore, it is possible that the association between physical workload and LBP might be overestimated because subjects with LBP may have overestimated their physical work load exposure compared with those without LBP. Results from other studies [41,42] have suggested that differential misclassification is not enough to bias the results substantially.

A part of the studied population consisted of participants without LBP experience but with experiences of musculoskeletal pain in the shoulders, neck or upper back. To our knowledge the prospective effect of neck, shoulder and upper back pain on LBP has not been investigated. However, a study by Juul-Kristensen et al. (2006) found that the prevalence of LBP was higher among female computer users with pain in the neck, shoulders or upper back compared with a control group without pain [43]. Another study of van Nieuwenhuyse et al. (2004) found that upper limb complaints increased the risk of LBP [39]. Therefore it could be argued that our study population had a higher risk of developing LBP compared with a study population without any musculoskeletal pain experience, which would give fear-avoidance beliefs and physical work load more strength than in a population without any musculoskeletal pain.

We believe that our categorization of LBP in four groups was useful in elucidating different relations with fear-avoidance beliefs. The results demonstrated that fear-avoidance was predictive for the group with > 30 days of LBP but not for the groups with only 1-7 and 8-30 days of LBP. This emphasizes the relevance of making subgroups when dealing with low back pain because future intervention programmes may be more effective if they aim to reduce fear-avoidance and LBP among those with relatively many days with LBP a year.

## Conclusion

The results from this study suggest that for those without previous LBP, physical work load is unrelated to the development of LBP, while fear-avoidance beliefs are prospectively related to episodes of LBP. For those with previous LBP, both physical work load and fear-avoidance beliefs are important for new episodes of LBP. Further, the results suggest that fear-avoidance beliefs are more strongly related to relatively many days of LBP than to few days of LBP. This supports the idea that fear-avoidance beliefs are present before the initiation of LBP and that fear-avoidance beliefs are developed in a reciprocal process with the LBP pain experience. From a treatment perspective, focusing on changing fear-avoidance beliefs among those with more or less chronic LBP may be beneficial. Health care professionals may benefit from additional education or information about how to cope with acute or chronic LBP. Particularly information about the potentially harmful effect of avoidance-behaviour could be useful.

## Abbreviations

LBP: low back pain; FABQ: fear-avoidance beliefs questionnaire; FAB-work: fear-avoidance beliefs about work; FAB-physical activity: fear-avoidance beliefs about physical activity.

## Competing interests

The authors declare that they have no competing interests.

## Authors' contributions

JNJ participated in the formulation of the study, performed the statistical analysis, drafted the manuscript and was responsible for the design of the questionnaire survey and data collection. KAL participated in the formulation of the study and participated in the description of background knowledge. KNN participated in the formulation of the study and choice of statistical methods. VBO participated in the formulation of the study and choice of statistical methods. All authors critically read, revised and finally approved the manuscript.

## Pre-publication history

The pre-publication history for this paper can be accessed here:


